# The Unique Biosynthetic Route from *Lupinus* β-Conglutin Gene to Blad

**DOI:** 10.1371/journal.pone.0008542

**Published:** 2010-01-06

**Authors:** Sara Monteiro, Regina Freitas, Baru T. Rajasekhar, Artur R. Teixeira, Ricardo B. Ferreira

**Affiliations:** 1 Instituto de Tecnologia Química e Biológica, Universidade Nova de Lisboa, Oeiras, Portugal; 2 Instituto Superior de Agronomia, Universidade Técnica de Lisboa, Lisboa, Portugal; Purdue University, United States of America

## Abstract

**Background:**

During seed germination, β-conglutin undergoes a major cycle of limited proteolysis in which many of its constituent subunits are processed into a 20 kDa polypeptide termed blad. Blad is the main component of a glycooligomer, accumulating exclusively in the cotyledons of *Lupinus* species, between days 4 and 12 after the onset of germination.

**Principal Findings:**

The sequence of the gene encoding β-conglutin precursor (1791 nucleotides) is reported. This gene, which shares 44 to 57% similarity and 20 to 37% identity with other vicilin-like protein genes, includes several features in common with these globulins, but also specific hallmarks. Most notable is the presence of an ubiquitin interacting motif (UIM), which possibly links the unique catabolic route of β-conglutin to the ubiquitin/proteasome proteolytic pathway.

**Significance:**

Blad forms through a unique route from and is a stable intermediary product of its precursor, β-conglutin, the major *Lupinus* seed storage protein. It is composed of 173 amino acid residues, is encoded by an intron-containing, internal fragment of the gene that codes for β-conglutin precursor (nucleotides 394 to 913) and exhibits an isoelectric point of 9.6 and a molecular mass of 20,404.85 Da. Consistent with its role as a storage protein, blad contains an extremely high proportion of the nitrogen-rich amino acids.

## Introduction

The seed storage proteins classified as 7S globulins occur in a wide range of plants and are often called vicilins because of their presence in the Viciae group of legumes [1]. Vicilin proteins have apparently evolved from the same precursor, the tandem duplicate of a single primordial gene. In addition, all vicilin proteins have the same underlying secondary, tertiary and quaternary structures. Canavalin and phaseolin, for example, exhibit three-dimensional structures that are extraordinarily similar [2]. Although a number of sequences have been reported for *Lupinus* seed storage proteins, including α- conglutin, γ-conglutin and δ-conglutin [3], the gene encoding the major *Lupinus* storage globulin, i.e. β-conglutin, has not yet been sequenced.

Storage protein deposition in maturing seeds and mobilization in germinating seeds are highly specialized processes. In vicilin metabolism, synthesis and degradation, the antagonistic processes of protein turnover, are temporarily separated, occurring during different developmental stages. Thus, no degradation of mature vicilins is observed at the time of synthesis and accumulation during seed maturation. Also, no synthesis of vicilins is detected when breakdown takes place at the time of germination and seedling growth [4]. This dilemma becomes even more complicated because vicilins are typically synthesized as precursors that undergo molecular maturation by limited proteolysis before deposition in the protein storage vacuoles (PSV), [4]. However, synthesis and maturation of vicilins are not apparently developmentally separated [5]. On the other hand, few proteins are capable of surviving the lytic environment of the PSV. Foreign (nonstorage proteins) and genetically modified storage proteins tend to be proteolytically unstable in PSVs and consequently often fail to accumulate or are fragmented when expressed in plants [6], [7]. Members of the vacuolar processing enzyme (VPE) family are most likely responsible for the specific polypeptide processing events of seed storage protein in PSV. These VPE family members have been associated with functions other than seed storage protein processing, including storage protein catabolism during germination, although these functions have not been demonstrated *in vivo*. Nevertheless, VPEs have been shown to constitute merely one pathway for processing seed storage proteins, with other proteolytic enzymes also processing storage proteins into chains capable of stable accumulation in mature seeds [8].

The vicilins show significant heterogeneity in the processing of the mature protein. Vicilins from cacao, cotton, walnut, French bean and soybean possess a hydrophobic signal sequence followed by a markedly hydrophilic region containing a varying number (from 2 to 6) of Cys-X-X-X-Cys motifs, which may or not be cleaved from the dominant mature protein [9], [10]. Glycosylation level is another post-translational chemical modification that contributes to vicilin microheterogeneity [9]. In addition, the pattern of degradation of the vicilin-type globulins during germination and seedling growth of legume species was found to be species specific and independent of the rate of proteolysis. In some cases, a transient accumulation of stable intermediates of vicilin catabolism is detected [11]. Thus, for example, a 20 kDa polypeptide accumulates abundantly in *Lupinus albus* cotyledons between days 4 and 12 after the onset of germination [12]. This polypeptide is a stable breakdown product of β-conglutin catabolism and has been shown to display lectin activity [13], an observation that highlights the potential physiological roles played by this protein. In this respect, vicilins have long been considered important seed storage proteins that not only play a role as a nutrient reserve for the germinating seedling [4] but also appear to serve a dual role as plant defense-related proteins. In cowpea, for example, vicilins have been shown to bind to chitin and thus to inhibit fungal and insect growth [14]–[16].

In this work, the sequence of β-conglutin precursor gene is presented and characterized. The unique biosynthetic route to the stable breakdown product of β-conglutin catabolism, herein termed blad, is described. Two major rounds of limited proteolysis were demonstrated. The nucleotide sequence of the gene fragment encoding blad was determined and located within the sequence of its precursor gene, i.e. β-conglutin precursor.

## Results

### The β-Conglutin Precursor Gene

β-Conglutin, a vicilin or 7S globulin, is the major seed storage protein in *Lupinus* species. β-Conglutin subunits appear to be synthesized in *Lupinus albus* developing cotyledons as a single glycosylated precursor polypeptide [17]. The complete sequence of the gene encoding the precursor of β-conglutin from *L. albus* was obtained by rapid amplification of cDNA ends (RACE) technique.

**Figure 1:**
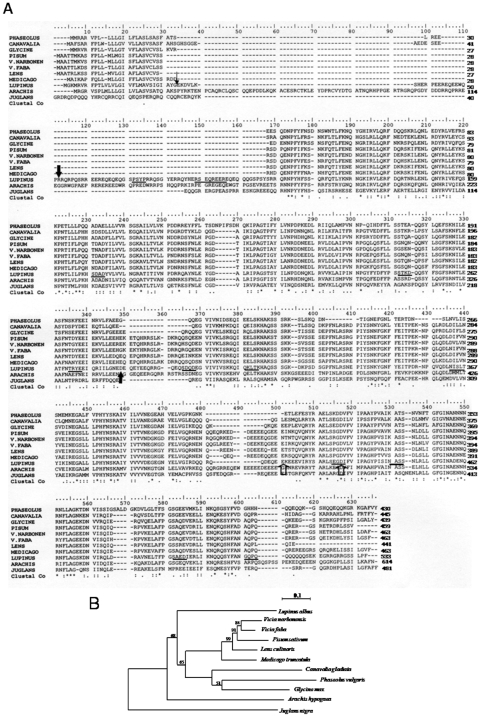
Comparison of β-conglutin precursor with the vicilins from other legume species. (A) Multiple sequence alignment of predicted amino acid sequences of *Lupinus albus* β-conglutin precursor with other legume vicilins. In the β-conglutin sequence, the small arrowhead designates the putative cleavage of the signal peptide; larger closed arrowheads denote the N- and C- terminals of blad; larger open arrowheads denote the beginning and the end of the UIM; potential *N*-glycosylation and phosphorylation sites are underlined by thick and thin lines, respectively. The asterisks indicate that the residues in column are identical in all sequences in the alignment; colons indicate conservative substitutions; full stops mean that semi-conserved substitutions are observed. Hyphens are inserted to optimize the alignments. LUPINUS- *Lupinus albus*; V.FABA- *Vicia faba*; LENS- *Lens culinaris*; PISUM- *Pisum sativum*; V.NARBORENSIS- *Vicia narborensis*, GLYCINE- *Glycine max*; PHASEOLUS- *Phaseolus vulgaris*; CANAVALIA- *Canavalia gladiata*; MEDICAGO- *Medicago truncatula*; JUGLANS- *Juglans nigra*; ARACHIS- *Arachis hypogaea*. (B) Phylogram of vicilin-like proteins with high homology to β-conglutin from *Lupinus albus*. The following proteins were included in the phylogenetic analyses: *Vicia faba* (vicilin precursor, CAA68525), *Lens culinaris* (allergen Len c, CAD87731), *Pisum sativum* (vicilin, CAA32239), *Vicia narbonensis* (vicilin precursor, CAA96514), *Glycine max* (alpha' subunit of beta-conglycinin, BAE02726), *Phaseolus vulgaris* (phaseolin, AAC04316), *Canavalia gladiata* (canavalin, CAA33172), *Medicago truncatula* (cupin, ABD28364), *Juglans nigra* (vicilin, AAM54366) and *Arachis hypogaea* (allergen Ara h 1, P43237).

The Swiss Prot protein database was searched using the FASTP program for sequences similar to β-conglutin from *Lupinus albus*. The legume vicilin sequences included in this analysis were selected according to their homology to β-conglutin. The multiple sequence alignment of the deduced amino acid sequence from *L. albus* β-conglutin precursor with those from other legume vicilins is shown in Figure 1A.

A comparative analysis was made between the β-conglutin precursor gene and genes encoding other vicilin-like proteins. The major features are (Figure 1A): an amino acid sequence similarity of 44 to 57% and an amino acid sequence identity of 20 to 37%; the presence of regions extremely rich in arginine (residues 52 to 145) and glutamine (residues 46 to 102 and 500 to 517); the existence of a charged hydrophilic domain that follows the hydrophobic N terminus that lacks the Cys-X-X-X-Cys motif repeat present in other members of the vicilin-like proteins; the presence, in common with other vicilin-like proteins, of a repeated sequence (residues 117 to 299 and 310 to 509) thought to have arisen through the duplication of a primordial gene; the presence, in common with other vicilin-like proteins, of a cupin domain (residues 122 to 249 and 332 to 494), (the cupin superfamily was identified by Dunwell in 1998 [18]) and named on the basis of the conserved beta-barrel fold); the existence of 51 strictly conserved residues present in all members of the vicilin-like family studied (marked with an * in figure 1A); finally, the presence, exclusively in β-conglutin, of an ubiquitin-interacting motif (UIM; residues 415 to 432).

The phylogram depicted in Figure 1B was generated through analysis of legume vicilin amino acid sequences by use of the ClustalW. The alignment was refined manually to maximize the match around the residues that form the cupin domain. A phylogenetic tree was generated by the Neighbor-Joining method [19] using the package of programs PHYLIP version 3.67 [20]. The DNA distance matrix was calculated from the data set according to the JTT and using the Protdist program. The tree was subsequently calculated using the Neighbor program and visualized using the Njplot program [21]. The reliability of individual branches in the tree was assessed from a consensus based on 1000 randomly generated trees [22] using the Seqboot, Protdist, Neighbor and Consense programs of PHYLIP package. Three different clusters may be observed. In spite of the best homology scores obtained with *Glycine max* alpha' subunit of beta-conglycinin, phylogenetically, β-conglutin is not closely related with none of the selected sequences. Vicilin sequences from the *Viciae* tribe are clustered, and vicilin from *Pisum sativum* is closely related to the vicilin precursor from *V. narbonensis*. Distance between branching points of *Pisum sativum* sequence and both of the studied *Vicias* are close to zero. The unclustered position of *Juglans nigra* vicilin can be easily explained due to the fact that this species does not belong to the Leguminosae family.

Out of 421 residues where at least two sequences overlap, 51 residues (12.1%) are common to all sequences and a further 91 (21.6%) are found in 9 of the 11 species considered. These are indicated in Figure 1A. Most of the replacements are conservative. In addition, relative to canavalin, the other proteins contain apparent sequence deletions and insertions, which may possibly have arisen by short tandem sequence duplications.

### The Unique Biosynthetic Route from β-Conglutin to Blad

**Figure 2:**
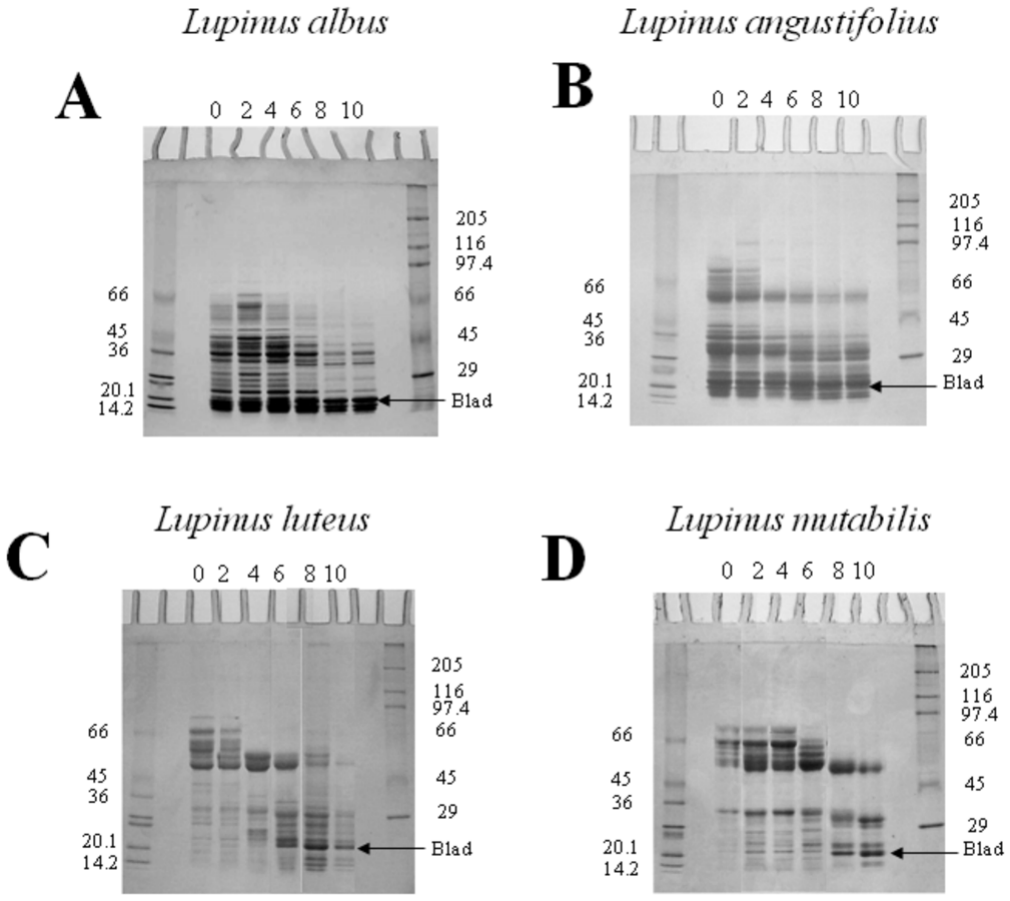
One-dimensional, structural analysis of β-conglutin from *Lupinus albus*, *Lupins angustifolius*, *Lupinus luteus* and *Lupinus mutabilis*. *L. albus* (A), *L. angustifolius* (B), *L. luteus* (C) and *L. mutabilis* (D) seeds were germinated for up to 10 days, and β-conglutin extracted, purified, analysed by one-dimensional SDS-PAGE and stained for total protein. Seeds were germinated for the number of days indicated on top of the gels. Fifty µg of protein were loaded in each lane. Molecular masses of standards are indicated in kDa.

In the case of *L. albus*, the one-dimensional SDS-PAGE analysis of β-conglutin revealed that the mature protein is composed of 10 to 12 major types of subunits, with molecular masses ranging from 15 to 72 kDa, as well as a considerable number of minor constituents (Figure 2A, lane 0). In contrast, the protein from *L. angustifolius* contains two groups of polypeptides: a heavier group (50 to 72 kDa) and more abundant, lighter group (15 to 40 kDa) (Figure 2B, lane 0), whereas β-conglutins from *L. luteus* and *L. mutabilis* are essentially composed by a heavy group of polypeptides (50 to 70 kDa; Figure 2C, D).

Despite the large heterogeneity observed among different *Lupinus* species in what concerns the polypeptide composition of β-conglutin, this protein suffers an identical fate during germination in all species examined (results not shown). Between days 3 and 5 following the onset of germination, β-conglutin undergoes a dramatic change in its structure and concentration, involving the appearance of a new set of polypeptides, including a higher molecular mass group, whose concentration steadily declines until complete disappearance after 11 days, and a lighter molecular mass group, whose concentration surprisingly increases from 5 to 11 days (Figure 2, and data not shown). Particularly evident is the abrupt accumulation of an abundant 20 kDa polypeptide, herein termed blad, in the cotyledons of all *Lupinus* species during the 4th day after imbibition, which is maintained in high amounts in these organs during the following days. Based on similar information, Ramos et al. (1997) [13] suggested that blad derives from β-conglutin subunits in *L. albus*.

**Figure 3:**
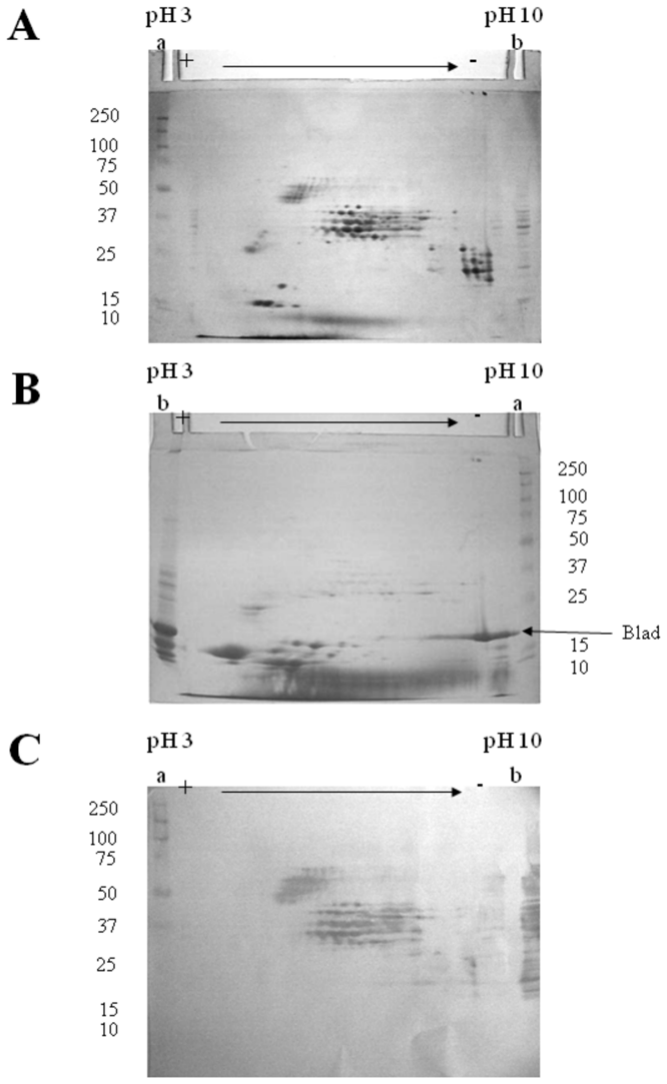
Two-dimensional, structural analysis of β-conglutin and blad from *Lupinus albus*. β-Conglutin (A,C) and the protein containing blad (B) were extracted, purified from the cotyledons of dry seeds and eight-days germinated seedlings, respectively, and subjected to two-dimensional electrophoresis. The gels were either stained for total protein (A,B) or transferred onto a membrane and probed with anti-blad antibodies (C). Two hundred µg of protein were used to prepare the 2D-gels (A,B) and 50 µg to prepare the 2D-blot (C). Lanes a: molecular mass standards (kDa) (precision protein standards prestained, broad range, Bio-Rad, in C). Lanes b: β-conglutin (A, 50 µg; C, 15 µg) or the native protein containing blad (B, 50 µg).

Analysis of β-conglutin from *L. albus* by two-dimensional electrophoresis allows the detection of a very large number of distinct polypeptides (Figure 3A). The majority of these polypeptides may be grouped into five distinct classes, which differ in their mass to charge ratio and which may be designated by increasing order of acidity: class I, containing basic polypeptides, with isoelectric points (pI) between 9 and 10 and molecular masses in the range of 20 to 30 kDa; class II, the major class, comprising neutral to moderately basic polypeptides, with pI values between 5.5 and 9 and molecular masses in the range 30 to 45 kDa; class III, composed of moderately acidic polypeptides, with pI values between 5 and 6 and molecular masses between 45 and 70 kDa; class IV, comprehending acidic polypeptides characterized by pI values between 4 and 5.5 and molecular masses of 15 to 20 kDa. Finally, class V includes acidic polypeptides with pI values of 4 to 5 and molecular masses of 25 to 35 kDa. Other polypeptides with molecular masses between 20 and 40 kDa and pI values between 8 and 9 are present in the 2-D gel, but their relation (if any) with the five major regions is unknown.

As expected, analysis of the native protein that contains blad by two-dimensional electrophoresis produces a polypeptide pattern that is totally different from that of the precursor protein (Figure 3B). Blad appears to be an extremely basic (pI between 9 and 10), homogeneous polypeptide. A considerable number (about 10) of small polypeptides are also present in the gel (pI from 3 to 6, molecular masses from 10 to 15 kDa), as well as minute amounts of the β-conglutin, class II polypeptides.

The use of polyclonal antibodies, produced specifically against blad, to probe a blot obtained from a gel identical to the one depicted in Figure 3A, clearly confirms that blad is derived from β-conglutin (Figure 3C). However, not all β-conglutin polypeptides serve as precursors for blad. Only the β-conglutin polypeptides belonging to classes II and III (Figures 3A and 3C) are precursors of blad.

**Figure 4:**
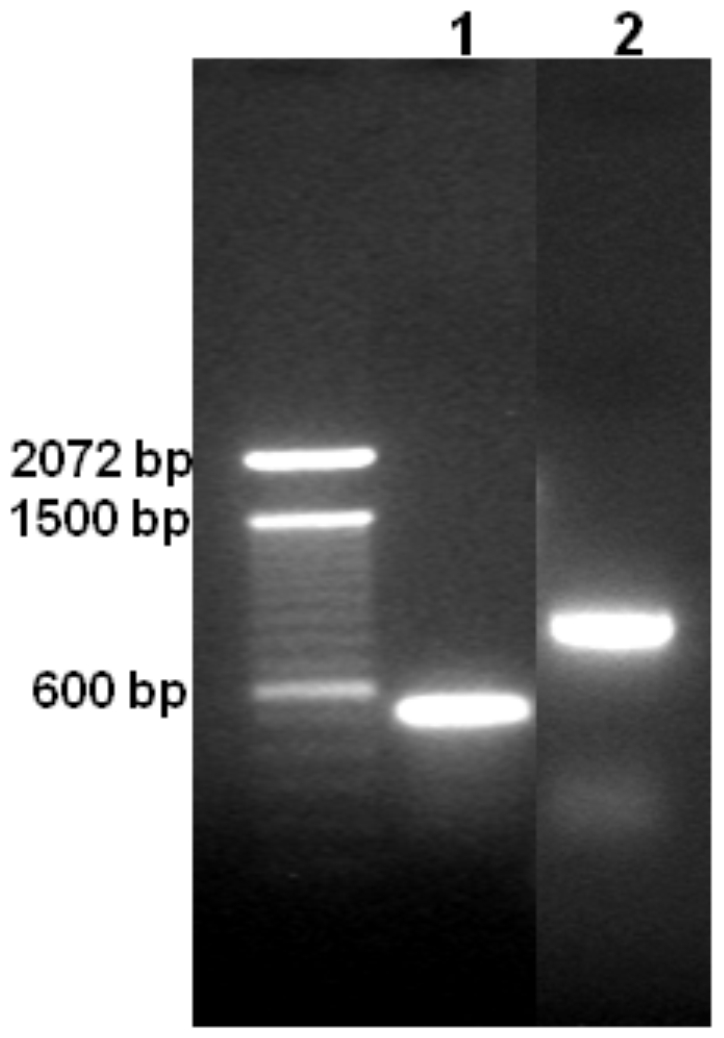
Agarose gel showing the products obtained when the mRNA fragment (lane 1) or the gene fragment (lane 2) were amplified using primers specific for both terminals of blad. The size of markers is indicated on the left.

Using pure blad, an attempt was made to obtain its complete amino acid sequence by conventional methods. Therefore, blad was subjected to N-terminal sequencing (NTS), C-terminal sequencing (CTS) and NTS after purification by reverse phase-HPLC of the peptide fragments obtained after enzymatic digestion of blad with trypsin (Tryp) or endoproteinases Lys-C, Asp-N or Glu-C. Chemical cleavage with cyanogen bromide (this reagent cleaves at methionyl residues) was not attempted because, in common with the proteins of other legume seeds, lupin seed proteins are nutritionally deficient, with lower than desirable levels of the sulphur-containing amino acids [23]. The results obtained are presented in Table 1 and show the NTS, the CTS and the amino acid composition of blad. The complete amino acid sequence of blad could then be determined after locating its N- and C-terminal sequences (marked with large closed arrowheads in Figure 1A) into the complete sequence of its precursor, β-conglutin. This result was confirmed by matching the sequences of all the peptide fragments obtained by NTS after enzymatic digestion of blad. The nucleotide sequence of the gene fragment encoding blad was further confirmed by amplification and sequencing of the product obtained using primers specific for both ends of blad within the nucleotide sequence of the mRNA encoding β-conglutin precursor (Figure 4, lane 1). When the same primers encoding blad terminals were used to amplify a sequence from genomic DNA, a 100 bp heavier product was obtained (figure 4, lane 2), indicating the presence of an intron in the gene fragment encoding blad.

**Table 1:** N-Terminal sequence, C-terminal sequence and amino acid composition of blad.

A.	N-Terminal sequence^a^	
	^1^RRQRNPYHFS SQRFQTLYXN GXGXXRVLERFDQRTNR^37^	
B.	C-Terminal sequence^b^	
	LXNXD	
C.	Amino acid composition^c^	
	Ala (7)	Leu (15)
	Arg (18)	Lys (7)
	Asn (17)	Met (0)
	Asp (10)	Phe (9)
	Cys (0)	Pro (9)
	Glu (8)	Ser (11)
	Gln (11)	Thr (10)
	Gly (7)	Trp (0)
	His (2)	Tyr (12)
	Ile (12)	Val (8)

a N-Terminal sequencing was obtained by Edman degradation of blad N-terminal or internal fragments obtained by enzymatic digestion of blad with trypsin or endoproteinases Lys-C, Asp-N or Glu-C and purified by reverse phase-HPLC. X represents an unidentified residue.

b C-Terminal sequencing was performed by the method of Boyd et al. (1992).

c Number of residues indicated in parenthesis. Total number of residues = 173.

Using the tool supplied by Gasteizer et al. [24] to calculate the estimated pI and molecular mass of the EMBL/GenBank accession number DQ142920 or the user-entered blad amino acid sequence, an estimated pI of 9.6 and a molecular mass of 20,404.85 Da were calculated for blad, values which are in very good agreement with those estimated by 2-D electrophoresis and SDS-PAGE, respectively. In addition, blad does not include any cysteine, methionine or tryptophan residues, but contains an extremely high proportion of the nitrogen-rich amino acids (Table 1).

## Discussion

β-Conglutin, the most abundant storage protein present in the cotyledons of *Lupinus* species, is a member of the 7 S family or vicilin family of storage proteins [3]. Surprisingly, the gene encoding this protein has not yet been sequenced. On the contrary, a number of sequences have been reported for the other *Lupinus* seed storage proteins, namely α-conglutin (accession number Q96475), γ-conglutin (Q9FSH9, Q9FEX1, Q958M6, L39786) and δ-conglutin (Q99235, P09930, P09931).

The mRNA encoding the precursor of *L. albus* β-conglutin was sequenced. This sequence, published under the NTrEMBL accession number Q6EBC1/GenBank accession number AY500372, was compared with the vicilin sequences of *Vicia faba* (vicilin precursor, CAA68525), *Lens culinaris* (allergen Len c, CAD87731), *Pisum sativum* (vicilin, CAA32239), *Vicia narbonensis* (vicilin precursor, CAA96514), *Glycine max* (alpha' subunit of beta-conglycinin, BAE02726), *Phaseolus vulgaris* (phaseolin, AAC04316), *Canavalia gladiata* (canavalin, CAA33172), *Medicago truncatula* (cupin, ABD28364), *Juglans nigra* (vicilin, AAM54366) and *Arachis hypogaea* (allergen Ara h 1, P43237).

Consistent with its role as a storage protein is the presence of regions extremely rich in arginine (residues 52 to 145) and glutamine (residues 46 to 102 and 500 to 517). Analysis of the amino acid sequence of β-conglutin shows that it shares 44 to 57% similarity and 20 to 37% identity with the vicilin-like seed storage proteins (Figure 1A). The vicilin-like family of proteins share several conserved features, and a detailed comparison of the β-conglutin sequence with other members of this family highlights the close relationship of the β-conglutin with this group of proteins.

Seed storage proteins typically contain a hydrophobic signal sequence that targets the newly synthesized polypeptides to the endoplasmic reticulum, where the protein undergoes a variety of modifications, including the proteolytic removal of the signal sequence, glycosylation, and sorting for transit through the remainder of the secretory pathway. The vicilin-like polypeptides can be roughly grouped into two size classes depending on the length of a variable highly charged segment that follows the hydrophobic N terminus [25]. Storage polypeptides containing this hydrophilic segment (which ranges in size from 90 to 183 residues) are approximately 70 kDa in size, whereas those lacking this region are approximately 50 kDa. In addition, the Cys-X-X-X-Cys motif that is repeated six times in this portion of the cotton vicilin and twice in the vicilin-like storage proteins of cocoa, barley, soybean, wheat, and maize, is absent in the β-conglutin sequence. Thus, β-conglutin shares the charged hydrophilic domain but not the Cys-X-X-X-Cys motif within this domain with other members of the vicilin-like proteins.

A third shared feature of β-conglutin with other vicilin-like proteins is the presence of a repeated sequence that is thought to have arisen through the duplication of a primordial gene [26]–[28]. The three-dimensional structures of both phaseolin and canavalin, vicilin-like proteins from *Phaseolus vulgaris* and *Canavalia ensiformis*, respectively, demonstrate that this duplicated sequence forms the basis for the symmetrically reiterated Swiss roll domain structure [2], [27], [29], [30]. Alignment of the β-conglutin sequence with the other vicilin-like proteins shows that β-conglutin contains a similar internal repeat (residues 117 to 299 and 310 to 509; Figure 1A).

Lawrence et al. [27] identified 26 strictly conserved residues present in all members of the vicilin-like proteins. Our results show 51 strictly conserved residues present in all members of the vicilin-like family studied (indicated by an * under the sequences in Figure 1A). Analysis of these conserved residues in the context of the known three-dimensional structures demonstrates their importance in maintaining the overall structure and intramonomer contacts [27]. As such, β-conglutin appears likely to contain similar tertiary motifs and to be organized in similar three-dimensional structures.

Finally, a most interesting motif that is exclusively present in β-conglutin emerged from the motif sequence profiling. Between residues 415 and 432, an ubiquitin interaction motif was found. The Ubiquitin Interacting Motif (UIM) was first described in the 26S proteasome subunit S5a [31]. The recognition of ubiquitylated proteins is frequently mediated by a number of conserved ubiquitin binding modules, including the UIM [31], [32]. In addition to the proteasomal S5a subunit, UIM occurs in a wide variety of proteins, either involved in ubiquitylation, ubiquitin metabolism or interaction with ubiquitin-like modifiers [32]. Among the UIM proteins are two different subgroups of the UBP family of deubiquitylating enzymes, one F-box protein, one family of HECT-containing ubiquitin-ligases (E3s) from plants, and several proteins containing ubiquitin-associated UBA and/or UBX domains [33]. UIMs are therefore particularly prevalent in proteins that function in the pathways of endocytosis and vacuolar protein sorting [32].

The recognizable part of this motif contains 20 residues, comprising a highly conserved Φ-X-X-Ala-X-X-X-Ser-X-X-Ac core, in which Φ denotes a large hydrophobic residue and ‘Ac’ denotes an acidic residue. This core region is preceded by a block of four preferentially acidic residues. Such a short sequence motif is unlikely to form an independent folding domain. Instead, based on the spacing of the conserved residues, the motif probably forms a short amphipathic α helix that can be embedded into different protein folds [32]. UIM peptides from several proteins involved in endocytosis and vacuolar protein sorting bind specifically, but with low affinity, to free ubiquitin. Full affinity ubiquitin binding requires the presence of conserved acidic patches at the N and C terminus of the UIM, as well as highly conserved central Ala and Ser residues [34].

In animals, the functions of several UIM-containing proteins suggest a role for this motif in the pathogenesis of neurodegeneration [32]. It was proposed that the UIM might be a general ubiquitin-binding motif. Although this role is well documented for the UIM in the proteasomal S5a subunit, a similar function for the UIM present in the factors involved in endocytosis and lysosomal degradation remains to be established.

It is rather difficult at the present stage of research to understand the presence of the UIM in β-conglutin. A direct involvement of the ubiquitin/proteasome pathway during germination and seedling growth of *Lupinus* seeds has been demonstrated [12]. It is tempting to speculate that the presence of the UIM explains the unique nature of β-conglutin catabolism (see below), possibly involving the ubiquitin/proteasome proteolytic system. As a whole, these data indicate that β-conglutin shares several conserved features with other vicilin-like proteins, but also exhibits some hallmarks of its own.

β-Conglutin subunits are synthesized in developing cotyledons as glycosylated precursor polypeptides, the number of which seems to vary with the species considered–apparently one in *L. albus* and *L. angustifolius*, and three to four in *L. luteus* [17], [35], [36]. These precursor propolypeptides have been shown to assemble into multimeric forms around 190 kDa [35]. Developing cotyledons of *L. albus* contain, 35 days after flowering, a major 64 kDa polypeptide which is the precursor of β-conglutin. The amount of this polypeptide decreases during seed maturation, without completely disappearing in the mature seed. The precursor oligomer of 190 kDa consists of an association of three 64 kDa subunits and contains covalently linked carbohydrate [17].

During cotyledonary development towards the mature seed, the β-conglutin precursor undergoes a cycle of intensive processing, involving proteolytic processing and changes in glycosylation. As a result, the precursor(s) give rise to a complex array of processed forms, due to a large number of processing sites, which are apparently assembled into trimers with a molecular mass off approximately 187 kDa [23]. This process is illustrated by the polypeptide composition of mature β-conglutin present in the dry seed. A simple one-dimensional SDS-PAGE analysis of β-conglutin (Figure 2A, lane 0) reveals that the mature protein is composed of 10 to 12 major types of subunits, with molecular masses ranging from 15 to 72 kDa, as well as a considerable number of minor constituents. Two-dimensional electrophoretic analysis of β-conglutin (Figure 3A) further indicates that the individual molecules of this storage molecule are assembled from an extraordinary large number of distinct polypeptides, with almost a continuum of polypeptides ranging in molecular mass from 15 to 72 kDa. Given the very large number of available different subunits and the reported molecular mass of 180 to 200 kDa for the native protein, it seems clear that β-conglutin is characterized by a rather broad microheterogeneity that appears so characteristic of this protein. This has been recognized for a considerable number of years [17], [23], [37]–[40].

During seed germination and subsequent plantlet growth, β-conglutin undergoes a cycle of intensive limited proteolysis. Moreover, despite the large heterogeneity detected among different *Lupinus* species in what concerns the polypeptide composition of β-conglutin (lanes 0 in Figure 2), this protein suffers an identical fate during germination in all *Lupinus* species examined. Between days 3 and 5 following the onset of germination, β-conglutin undergoes a dramatic change in its structure and concentration, involving the appearance of a new set of polypeptides, including a higher molecular mass group, whose concentration steadily declines until complete disappearance after 11 days, and a lighter molecular mass group, whose concentration surprisingly increases from 5 to 11 days. Particularly evident is the abrupt accumulation of an abundant 20 kDa polypeptide (herein referred to as blad) in the cotyledons of all *Lupinus* species during the 4^th^ day after imbibition, and which is maintained in high amounts in these organs, rapidly declining after about 12 to 14 days [12], [13]. The native protein containing blad exhibits an apparent molecular mass of 210 kDa (when estimated by gel filtration), being composed of a major 20 kDa polypeptide (blad) and of at least three minor lower molecular mass polypeptides and several minor higher molecular mass polypeptides, when assayed by one-dimensional SDS-PAGE. In this way, this round of intensive limited proteolysis, several tens of β-conglutin constituent subunits are converted into the homogeneous, highly basic blad (Figure 3), which may therefore be considered as a stable, intermediary product of β-conglutin catabolism.

A close inspection of Figure 3C could suggest the presence of small amounts of blad in dry lupin seed cotyledons. However, it has been clearly demonstrated in some of our previous articles that blad starts to accumulate in lupin cotyledons around 4 days after the onset of germination [11], [13], [39]. Thus, the band with a molecular mass of approximately 20 kDa that is visible in Figure 3C corresponds to the acidic polypeptide of α-conglutin [38], [39].

A controversial difference occurs between the molecular mass generally considered in the literature for β-conglutin (180–200 kDa) and the apparent native molecular mass estimated by FPLC gel filtration on a Superose column for the native protein containing blad as 210 kDa. How can a precursor protein undergo a catabolic process and originate a larger product that transiently accumulates in *Lupinus* cotyledons. It is possible that subunit rearrangement occurs during this transition. Alternatively, the lectin activity of blad may have lead to an underestimation of the molecular mass of the native protein containing blad, due to a possible interaction between blad and the gel filtration matrix.

As referred above, blad exhibits an identical pattern of synthesis and accumulation in all four *Lupinus* species studied. However, among several other legume species examined (*Glycine max*, *Pisum sativum*, *Vicia faba*, *Vicia sativa*, *Lathyrus cicera*, *Lathyrus sativus* and *Arachis hypogaea*) no pattern in the catabolism of storage proteins was detected that resembled that of β-conglutin. In other words, no polypeptide was found to accumulate transiently during germination and plantlet growth of those legume species [11]. It seems therefore that blad is unique to the *Lupinus* genus.

Extensive purification of blad and sequencing of its N- and C- terminals, as well as of several internal sequences by conventional methodologies allowed the precise location of the nucleotide sequence encoding blad within that of its precursor (Figure 1). This nucleotide sequence was further confirmed by amplification and sequencing of the product obtained using primers for both ends of blad within the nucleotide sequence of the mRNA encoding β-conglutin precursor. A detailed analysis of the nucleotide sequence corresponding to the gene fragment encoding blad, as well as of the corresponding amino acid sequence, revealed a number of noteworthy features: (i) the presence of an intron within the gene fragment encoding blad. (ii) a comparison of blad sequence (deposited in GenBank under the accession number DQ142920) with those of other proteins revealed no significant homology to any other published sequence. The long N-terminal sequence obtained by Edman degradation, together with the N-terminal sequences of several internal fragments, exhibit a high homology with the α′-subunit of β-conglycinin from *Glycine max*. It is interesting to note the occurrence of several intersubstitutions between glutaminyl (Q) and lysil (K) residues when the sequences of these two polypeptides are compared. However, the N-terminal sequences of other internal fragments as well as the C-terminal sequence gave no homology when compared to the sequences of β-conglycinin or vicilins from other species (Figure 1). (iii) A number of phosphorylation sites were predicted in blad sequence, consistent with the observation that blad is indeed phosphorylated (data not shown). (iv) An estimated pI of 9.6 and a molecular mass of 20,404.85 Da. (v) the amino acid composition of blad is not well balanced, mostly due to a complete absence of tryptophan, cysteine and methionine residues (Table 2). The lack of sulphur-containing amino acids comes at no surprise, if we take into account that legume seed storage proteins are notoriously poor in these amino acids. Furthermore, blad precursor, β-conglutin, represents the most deficient of the *Lupinus* storage proteins in sulphur amino acids [41], [42]. However, consistent with its role as a storage protein, blad contains an extremely high proportion of the nitrogen-rich amino acids, notably arginine (18 residues out of 173), asparagine (17 residues), glutamine (11 residues) and lysine (7 residues) (Figure 1).

**Table 2:** Sequence of the primers used to sequence the cDNA encoding blad and the β-conglutin precursor.

A.	Degenerate primers
	^5′^CAR CGI AAY CCI TAY CAY TTY AAY^3′^ (Fw)
	^5′^CAT TTC AAC TCT CAA AGG TTC^3′^ (Fw)
	^5′^CAI TTY AAY AGI CAR CGI TTY CAR^3′^ (Rw)
B.	Nondegenerate primers
	^5′^CTG ATG CTG ACT ACG TCC TCG TT^3′^ (Fw)
	^5′^TAT GGC GAT GCT CTC AGA ATC CCA^3′^ (Fw)
	^5′^ACC TCT CGA CAC GG^3′^ (Rw)
	^5′^ACC TCT CGA GCA CAC GG^3′^ (Rw)
C.	RACE primers
	^5′^ RACE Adapter ^5′^GCU GAU GGC GAU GAA UGA ACA CUG CGU UUG CUG GCU UUG AUG AAA^3′^
	^3′^ RACE Adapter ^5′^GCG AGC ACA GAA TTA ATA CGA CTC ACT ATA GGT_12_VN^3′^
	^5′^ RACE Outer primer ^5′^GCT GAT GGC GAT GAA TGA ACA CTG^3′^
	^5′^ RACE Inner primer ^5′^CGC GGA TCC GAA CAC TGC GTT TGC GTT TGA TG^3′^
	^3′^ RACE Outer primer ^5′^GCG AGC ACA GAA TTA ATA CGA CT^3′^
	^3′^ RACE Inner primer ^5′^CGC GGA TCC GAA TTA ATA CGA CTC ACT ATA GG^3′^

Considering the precise and characteristic pattern of accumulation of blad during *Lupinus* plantlet development, its very high abundance in cotyledons (it comprises the majority of the total cotyledonary protein in 8-day old plantlets–results not shown), its particular amino acid composition and its link to β-conglutin, there is no doubt that blad fulfills, as β-conglutin, an important role as a seed storage globulin. In particular, the atypical pattern of blad occurrence, exclusively during a very limited period of time in the life cycle of *Lupinus* plants, suggests that this protein may display other physiological roles, a subject that is currently under investigation.

## Materials and Methods

### Biological Material and Growth Conditions

Dry seeds of white lupin (*Lupinus albus* L.) cv. Leblanc, of blue lupin (*Lupinus angustifolius* L.) cv. Unicrop, of yellow lupin (*Lupinus luteus* L.) cv. ST80, and of pearl lupin (*Lupinus mutabili*s Sweet) cv. Potosi were obtained from a local market or kindly supplied by Dr. J.N. Martins (ISA, Lisboa, Portugal). When appropriate, the seeds were germinated for periods up to 10 days [39]. In all cases, the seed coats were removed and the intact cotyledons dissected from the axes and stored frozen at −80°C until needed.

In the experiments involving developing cotyledons, *L. albus* was grown under field conditions, between March and June (photoperiod of 12 to 15 h; air temperature of 15 to 22°C), at the Instituto Superior de Agronomia (Lisboa, Portugal). Flowers, cotyledons and intact pods were collected during pod development, from the flower to the mature, dry pod.

### Purification of Proteins

#### Purification of total globulins

The protein studies presented in this work use for the most part purified globulins, a relatively heterogeneous sample essentially composed of α-, β- and γ- conglutins, the three major seed storage proteins of *Lupinus* species. Comparison with total seed extracts that show that purification takes place during globulin isolation have been extensively preformed previously [43]. Thus, total seed extracts have been compared with total globulin extracts [43]; total seed extracts and total globulin extracts have been followed during *Lupinus* germination and seedling growth [12], [39]; the polypeptide pattern of each individual conglutin has been analysed during *Lupinus* germination and seedling growth [39].

Total globulins from *Lupinus* dry seeds were extracted and isolated as described by Santos et al. [43] The globulins were subsequently precipitated by the addition of ammonium sulphate (561 g/L), centrifuged at 30,000 *g* for 20 min at 4°C, resuspended in 50 mM Tris-HCl buffer, pH 7.5 (5.7 mL/g of cotyledon) and desalted on PD-10 columns previously equilibrated in the same buffer.

The cotyledons from germinated seedlings were ground and homogeneized with a mortar and pestle in water (pH adjusted to 8.0) containing 10 mM CaCl_2_ and 10 mM MgCl_2_ (2 mL/g fresh weight). The homogenate was incubated at 4°C for 30 min with agitation, filtered through cheesecloth and centrifuged at 30,000 *g* for 1 h at 4°C. The precipitate was suspended in the globulin solubilising buffer (2 mL/g fresh weight; 100 mM Tris-HCl buffer, pH 7.5, containing 10% (w/v) NaCl, 10 mM EDTA- and 10 mM EGTA and agitated during 30 min at 4°C. The globulin containing solution was centrifuged for 1 h at 30,000 *g* and 4°C and the resulting supernatant desalted on PD-10 columns (GE Healthcare Life Sciences; disposable desalting Sephadex G-25 Medium columns, 9.1 mL bed volume) previously equilibrated in 50 mM Tris-HCl buffer, pH 7.5.

#### Purification of β–Conglutin from Lupinus albus seeds

β-Conglutin, the main storage protein present in *Lupinus* seeds, was extracted and purified from either dry seeds of *Lupinus* species or from seeds germinated for up to 10 days. After obtaining the total globulin fraction as explained above, the individual globulins were fractionated and purified by FPLC anion exchange chromatography on a Q-Sepharose column ((GE Healthcare Life Sciences; ∅Ø = 1 cm; h = 8 cm; flow rate = 1.5 mL/min) essentially as described by Ferreira et al. [39]. The bound proteins were eluted with a gradient of NaCl (0 to 1 M) and desalted into 50 mM Tris-HCl buffer, pH 7.5. α- and γ-conglutins were purified from *L. albus* cotyledons as reported by [43].

#### Purification of the native protein containing Blad

Blad polypeptide was initially discovered in our laboratories in the 90 s. It was given a nickname based on the initials of the Portuguese words “banda de *Lupinus albus* doce”, meaning band from sweet *Lupinus albus*. The native protein containing blad, the 20 kDa, lectin-like polypeptide which is a breakdown product of β-conglutin catabolism, was extracted and isolated from the cotyledons of eight-days old seedlings. Following isolation of the total globulin fraction, precipitation with ammonium sulphate and desalting, performed as described above, the protein corresponding to β-conglutin was purified by FPLC anion exchange chromatography followed by FPLC gel filtration chromatography as follows: the total globulin fraction was loaded on the Q-Sepharose column (∅Ø = 1 cm; h = 8 cm; flow rate = 1.5 mL/min) previously equilibrated in 20 mM Tris-HCl buffer, pH 7.5, and eluted with a linear gradient of NaCl (0 to 1 M). The fraction containing the 20-kDa polypeptide, eluted between 0.25 and 0.35 M NaCl, was subjected to gel filtration on the FPLC Superose 12 HR 10/30 column (GE Healthcare Life Sciences), equilibrated in 0.1 M Tris-HCl buffer (pH 7.5). This last purification step does not affect the polypeptide pattern of the protein, but removes unidentified low molecular mass compounds that appear to interfere with the protein lectin activity (results not shown).

### Production of Anti-Blad Polyclonal Antibodies

Blad previously purified by the standard procedure described above, was subjected to preparative SDS-PAGE (10% w/v acrylamide). Total polypeptides were visualized with CuCl_2_ (negative staining; copper staining; [44]. The band corresponding to blad was sliced and the protein eluted as described before [38], desalted on a PD-10 column previously equilibrated with water and utilized to immunize two-month-old, male Wistar rats. Each rat was injected subcutaneously with 0.4 mL of a solution containing 0.125 mg blad and 0.2 ml complete Freund's adjuvant. To obtain a high titre, three identical booster injections were given every four weeks in complete Freund's diluted 1∶10 with incomplete adjuvant. At intervals, blood was collected from the heart and the titre followed by immunoblotting. Total blood was taken from the heart 9 days after the third booster injection. Blood samples were allowed to clot and the serum was collected and stored frozen at −80°C. Antibody specificity was thoroughly assessed [11].

### Electrophoresis and Immunoblotting

One-dimensional, sodium dodecyl sulphate-polyacrylamide gel electrophoresis (SDS-PAGE), western blotting and immunoblotting were performed as described before [45].

Two-dimensional electrophoresis (IEF/SDS-PAGE) was carried out as follows. Isoelectric focusing was performed using the IPGphor system (Amersham Pharmacia). Immobiline Drystrip gel strips (IPG strips) (13 cm, pH 3–10) were obtained from Amersham Pharmacia. IPG strips were rehydrated with 250 µL of a solution containing 0.5% (v/v) IPG-buffer pH 3–10, 7 M urea, 2 M thiourea, 2% (v/v) NP-40, 1% (v/v) dithiothreitol and protein sample in the IPGphor strip holders. The program utilized for isoelectric focusing included the following steps: rehydration −30 V, 12 h; step 1 - 200 V, 1 h; step 2 - 500 V, 2 h; step 3 - 1000 V, 2 h; step 4 - 8000 V, 3.5 h. After focusing, the gel strips were immediately frozen at −80°C. The second dimension (SDS-PAGE) was performed as described above except that the gel contained only the separating gel. The gel strips were thawed and equilibrated for 15 min, with agitation, in 50 mM Tris-HCl buffer, pH 8.8, containing 6 M urea, 26% (v/v) glycerol, 2% (w/v) SDS and 1% (w/v) dithiothreitol. The strips were subsequently equilibrated for another 15 min, with agitation, in a similar solution that contained 2.5% (w/v) iodoacetamide instead of the dithiothreitol, placed on top of the SDS-PAGE gel, sealed with 0.5% (w/v) agarose and electrophoresed (220 V, 15 mA for 15 min followed by 220 V, 30 mA).

The preparation of immunoblots from the 2D-gels was performed as described above.

For agarose gel electrophoresis, after PCR amplifications (see below), 10 µL of each amplification product was fractionated on 1.0% (w/v) agarose gels in TAE buffer (40 mM Tris acetate pH 8.5, 2 mM Na_2_EDTA.2H_2_O). The gels were stained with ethidium bromide (0.5 ng/mL) and the UV illuminated gel images were digitally captured with a CCD–camera from Gel Doc™ 1000 single wavelength Mini-Transilluminator (Bio-Rad) and acquired using Quantity One^R^ version 4.01 program (Bio-Rad).

### Polypeptide Sequencing

For the N-terminal sequencing, the polypeptide was immobilized on a ProBlot polyvinylidene difluoride polymer (PVDF) membrane from Applied Biosystems and sequenced by Edman degradation as described before [45], in a protein sequencer from Perkin-Elmer-Applied Biosystems (model 477A) on-line with an RP-HPLC unit (model 120A) for the identification of the step-wise released PTH-amino acids.

For the C-terminal sequencing, the polypeptide was immobilized on a membrane and subjected to C-terminal analysis essentially by the method proposed by Boyd et al. [46], using an Applied Biosystems sequencer on-line with an RP-HPLC unit for the identification of the step-wise released ATH- amino acids.

For determining the internal sequences of the polypeptide, i.e. the sequences of amino acid residues of the polypeptide other than its N- or C-terminal sequences, the polypeptide was immobilized on an Immobilon-P (Millipore) membrane. N-terminal sequence analysis of the peptides obtained was then performed as described above, following enzymatic digestion of the polypeptide and peptide purification from the polypeptide digest by RP-HPLC. Digestion of the polypeptide was achieved using a number of different proteases (obtained from Boehringer Mannheim): trypsin (EC 3.4.21.4) from bovine pancreas; endoproteinase Lys-C (EC 3.4.21.50) from *Lysobacter enzymogenes*; endoproteinase Asp-N (EC 3.4.24.33) from a mutant of *Pseudomonas fragi*; and endoproteinase Glu-C or protease V8 (EC 3.4.21.19) from *Staphylococcus aureus* V8.

Protein sequence similarity was searched at TIGR, EMBL-EBI and NCBI with the BLAST 2.0, BLAST tm, BLAST tx and BLAST tp algorithms.

### Polynucleotide Sequencing

Reverse Transcriptase-Polymerase Chain Reaction (PCR) products were purified using the High-Pure PCR Product Purification Kit (Roche). These polynucleotides were subsequently sequenced with Big-Dye Terminator v1-1 kit (Applied Biosystems) and analysed by multi-cappilary electrophoresis with ABI PRISM 3700 DNA Analyzer at STAB VIDA (Instituto de Tecnologia Química e Biológica, Oeiras, Portugal).

DNA sequence similarity was searched at TIGR, EMBL-EBI and NCBI with the BLAST 2.0, BLAST tm, BLAST tx and BLAST tp algorithms.

### Sequencing of the cDNA Encoding β-Conglutin Precursor

The cDNA encoding β-conglutin precursor was sequenced as outlined in Figure 1. Total RNA was extracted from developing *L. albus* seeds at the development stage corresponding to β-conglutin maximal synthetic rate [12]. The seed samples (100 mg) were ground in liquid nitrogen and the RNA was isolated using the Invisorb Spin Plant-RNA Mini Kit (Invitek), according to the manufacturer's specifications. The RNA was DNase treated using the RQ1 RNase-Free DNase (Promega). The RNA quality and quantity were checked by 1.0% (w/v) agarose gel electrophoresis, stained with ethidium bromide.

Single-stranded complementary DNA (cDNA) was synthesized using Cloned AMV Reverse Transcriptase (Invitrogen) according to the manufacturer's protocol. Approximately 250 ng of total RNA was used, in combination with 0.5 µg Oligo(dT)_20_ anchored (Invitrogen) as primer, 10 mM dNTP mix (Invitrogen) and H_2_O, and preheated at 65°C for 5 min to denature secondary structures. The mixture was then cooled rapidly in ice. The RNA was reverse transcribed in a final volume of 20 µL using 15 U AMV Reverse Transcriptase, 40 U RNaseOUT™ (Invitrogen), 10 mM dithiothreitol (DTT), 4 µL 5×First-Strand buffer, at 42°C for 50 min, and then stopped by heating at 70°C for 15 min. The RT mixture was incubated with 2 U Ribonuclease H (Invitrogen) at 37°C for 20 min and the cDNA stock stored at −20°C.

A fragment of the cDNA encoding β-conglutin precursor was specifically amplified by PCR, using degenerate oligonucleotide primers designed from the N-terminal and internal amino acid sequences deduced from blad by Edman degradation (see under “Polypeptide Sequencing”). The degenerate primers employed are shown in Table 2. PCR was performed in a Mastercycler gradient PCR machine (Eppendorf) using 1 µL of the cDNA, 50 ng of each oligonucleotide primer, 200 µM of dNTPs (Invitrogen), 2 U of FastStart Taq DNA Polymerase (Roche) and 5 µL 10× PCR buffer, in a 50 µL volume. The PCR protocol was 94°C denaturation step for 2 min, followed by 30 s at 52°C and 1 min at 72°C. It continued then with 35 cycles and a final elongation step at 72°C for 10 min.

The fragment of the cDNA encoding β-conglutin precursor was sequenced and the information obtained used to sequence the entire cDNA by the Rapid Amplification of cDNA Ends (RACE) technique. Using nondegenerate primers (Table 2) and RACE primers provided by the FirstChoice™ RLM-RACE kit (Ambion), the nucleotide sequence was deduced between the fragment and the 3′ end of the cDNA encoding β-conglutin precursor. The TOPO TA cloning kit (Invitrogen) was employed to clone the cDNA fragment in a suitable vector (PCR II-TOPO plasmid), which was then used to transform competent *Escherichia coli* cells (One Shot Top 10 Chemically Competent *E. coli*). The bacteria transformed with the cDNA fragment were selected by a 37°C, overnight incubation on a solid Luria-Bertani (LB) medium containing kanamycin. The selected bacteria were subcultured into liquid LB medium containing kanamicin and incubated overnight at 37°C. The grown bacteria colonies were subsequently used for plasmid purification by the Jetstar kit (Genomed). The restriction enzyme Eco RI (Invitrogen) was utilized to split the plasmid in two fractions: one comprising the majority of the plasmid DNA, and the other, considerably smaller, containing the cloned cDNA fragment. This fragment was sequenced and corresponds to the encoding sequence between the initial cDNA and the 3′ end of the cDNA encoding β-conglutin precursor. Based on this sequence, a new set of primers were designed (Table 2) and the same methodology was applied to complete the sequence of the cDNA encoding β-conglutin precursor, i.e. to sequence its 5′ terminal.

The nucleotide sequence of blad was deduced by positioning, in the nucleotide sequence of its precursor (i.e. the β-conglutin precursor), both the N-terminal and the C-terminal amino acid residue sequences obtained by polypeptide sequencing (see above).

### Neighbor-Joining Phylogenetic Tree

A phylogenetic tree was generated by the Neighbor-Joining method [19] using the package of programs PHYLIP version 3.67 [20]. The DNA distance matrix was calculated from the data set according to the JTT and using the Protdist program. The tree was subsequently calculated using the Neighbor program and visualized using the Njplot program [21].

The reliability of individual branches in the tree was assessed from a consensus based on 1000 randomly generated trees [22] using the Seqboot, Protdist, Neighbor and Consense programs of PHYLIP package.

### General Assays

Protein content was measured using a modification of the Lowry method [47].
